# *Staphylococcus aureus* Surface Protein SdrE Binds Complement Regulator Factor H as an Immune Evasion Tactic

**DOI:** 10.1371/journal.pone.0038407

**Published:** 2012-05-31

**Authors:** Julia A. Sharp, Charlene G. Echague, Pamela S. Hair, Michael D. Ward, Julius O. Nyalwidhe, Joan A. Geoghegan, Timothy J. Foster, Kenji M. Cunnion

**Affiliations:** 1 Department of Pediatrics, Eastern Virginia Medical School, Norfolk, Virginia, United States of America; 2 Department of Microbiology and Molecular Cell Biology, Eastern Virginia Medical School, Norfolk, Virginia, United States of America; 3 Microbiology Department, Moyne Institute of Preventive Medicine, Trinity College, Dublin, Ireland; 4 Children's Specialty Group, Norfolk, Virginia, United States of America; 5 Children's Hospital of The King's Daughters, Norfolk, Virginia, United States of America; University of Kentucky College of Medicine, United States of America

## Abstract

Similar to other highly successful invasive bacterial pathogens, *Staphylococcus aureus* recruits the complement regulatory protein factor H (fH) to its surface to inhibit the alternative pathway of complement. Here, we report the identification of the surface-associated protein SdrE as a fH-binding protein using purified fH overlay of *S. aureus* fractionated cell wall proteins and fH cross-linking to *S. aureus* followed by mass spectrometry. Studies using recombinant SdrE revealed that rSdrE bound significant fH whether from serum or as a purified form, in both a time- and dose-dependent manner. Furthermore, rSdrE-bound fH exhibited cofactor functionality for factor I (fI)-mediated cleavage of C3b to iC3b which correlated positively with increasing amounts of fH. Expression of SdrE on the surface of the surrogate bacterium *Lactococcus lactis* enhanced recruitment of fH which resulted in increased iC3b generation. Moreover, surface expression of SdrE led to a reduction in C3-fragment deposition, less C5a generation, and reduced killing by polymorphonuclear cells. Thus, we report the first identification of a *S. aureus* protein associated with the staphylococcal surface that binds factor H as an immune evasion mechanism.

## Introduction

Invasive pathogenic organisms infect and replicate in the host leading to symptomatic disease. Their success, however, depends on their ability to withstand the hostile environment of their host, i.e., the immune system. The complement cascade is central to host innate immune defenses and is activated within seconds of the microbe's entry [1]. The complement system comprises more than 30 proteins and operates in a catalytic cascade. Complement activation leads to opsonization, pro-inflammatory anaphylatoxin generation, and eventual assembly of the membrane attack complex [2], [3], [4], [5].

The three pathways of complement converge at the generation of C3-convertases which cleave C3 into the anaphylatoxin C3a and the potent opsonin C3b. While both the classical and lectin pathways are specific in their activation, the alternative pathway can be spontaneously activated without a recognition trigger [4], and is crucial for amplification of the complement cascade [5]. Due to the potent nature of this system, both membrane-bound and fluid-phase regulators are in place to protect host cells from complement attack.

The major fluid-phase complement regulator Factor H is a 155 kDa plasma protein that accelerates the decay of the alternative pathway C3 convertase, C3bBb. In addition to this important function, fH is a cofactor for factor I-mediated cleavage of C3b to iC3b [6], [7]. Factor H is abundantly present in plasma at approximately 500 µg mL, but this may vary due to environmental and genetic factors with a range of 116–562 µg mL reported [4], [8]. Due to its relatively high concentration and important regulatory functions, fH is a prime target for sequestration by pathogens where surface-bound fH can benefit their survival. As such, invasive human pathogens are known to take advantage of soluble regulators such as fH to evade the immune response. Examples of these include *Streptococcus pyogenes* and *Borrelia burgdorferi* which acquire fH via the M protein and Complement Regulator Acquiring Surface Proteins (CRASPs), respectively [9], [10], [11], [12], and *S. pneumoniae* which binds fH via the Pneumococcal Surface Protein C (PspC) [13], [14].

*S. aureus* is known to recruit the complement regulator factor I (fI) via cell wall component clumping factor A (ClfA) [15], [16]. As a master of complement evasion, *S. aureus* also secretes several proteins to dampen complement attack. Secreted *S. aureus* proteins such as the staphylococcal complement inhibitor (SCIN), extracellular complement-binding protein (Ecb) and extracellular fibrinogen-binding protein (Efb) can block convertase function, a crucial phase of the complement cascade [17], [18], [19], [20], [21], [22]. The staphylococcal superantigen-like 7 protein prevents generation of the anaphylatoxin C5a [23], while chemotaxis inhibitory protein of *S. aureus* (CHIPS) blocks the C5a receptor thereby impeding migration of neutrophils to the site of infection [24].

We have recently shown that *S. aureus* binds the complement regulatory protein fH to its surface to disrupt the alternative pathway convertase [25]. Here we show that *S. aureus* binds fH via the surface protein SdrE and that fH remains functionally active when bound to recombinant SdrE. Further, we demonstrate that SdrE provides a survival advantage when expressed on the surface of a surrogate bacterium.

## Methods

### Ethics Statement

Human blood was obtained from four healthy volunteers for generating serum used as a reagent in these studies. Eastern Virginia Medical School IRB approved this study protocol: 02-06-EX-0216. Written informed consent was provided by study participants.

### Bacteria

*S. aureus* strain Reynolds was grown to mid-logarithmic phase (OD600 0.8–1.5) in Columbia 2% NaCl broth at 37°C with shaking. *Escherichia coli* strains were grown at 37°C with shaking in Luria broth supplemented with 100 µg ampicillin/mL. *Lactococcus lactis* cells that constitutively express the staphylococcal protein SdrE or that contain the empty vector pKS80, as previously described [26], were grown in M17 broth containing 0.5% glucose and 5 µg/mL erythromycin at 30°C without shaking. *L. lactis* surface expression of SdrE was confirmed by immunoblotting.

### Buffers

GVBS-EDTA (veronal-buffered saline [VBS] with 0.1% gelatin and 0.01 M EDTA); raffinose buffer (5 mM Tris, 20 mM MgCl, 30% raffinose); GVBS ^++^ (VBS with 0.1% gelatin, 0.15 mM CaCl_2_, and 1.0 mM MgCl_2_), Mg-EGTA-GVBS (GVBS with 5 mM MgCl_2_ and 8 mM EGTA), and 60% DGVBS ^++^ (GVBS ^++^ with 3% dextrose).

### Serum and factor H

Normal human serum (NHS) was prepared as previously described [27]. The serum was pooled, aliquoted, and stored at −80°C. Heat-inactivated serum (HI-serum) was prepared by heating NHS at 56°C for 30 minutes. Purified fH was purchased from CompTech.

### Cell wall preparations

Cell wall extracts were prepared as described previously [28]. Briefly, cells from 20 ml of *S. aureus* cultures were washed twice with GVBS-EDTA and resuspended in 30% raffinose buffer for bacterial protoplast stabilization. DNase and protease inhibitors (Complete Mini, Roche) were then added. Cell wall proteins were extracted using 10 µg lysostaphin (Sigma) at 37°C for 1 hour, with rotation. The protoplasts were pelleted and cell wall proteins were recovered in the supernatant.

### Cell wall fractionation

*S. aureus* cell wall proteins were separated by size-exclusion chromatography and ionic exchange chromatography, as described by Hair et al. [16]. Briefly, size-exclusion chromatography was performed using a HiPrep 16/60 Sephacryl column (GE Healthcare) with 5- mL fractions collected at a rate of 1 mL/min in PBS. For ionic exchange chromatography, a 5-mL HiTrap Q HP column (GE Healthcare) was used with fractions eluted using a gradient of 1 M NaCl in 20 mM Tris buffer.

### Dot-blot detection of factor-H binding fraction and factor-H overlay blot

Column fractions were added to dot-blot wells to allow proteins to adhere to a PVDF membrane. Following blocking with 3% BSA in Tris-buffered saline with 0.1% Tween (TBST), the membrane was incubated with purified fH (20 µg fH/10 ml block buffer) overnight at 4°C, washed, then probed with chicken anti-fH antibody (1∶1000, Accurate Chemical) followed by anti-chicken HRP-labeled antibody (1∶1000, Genway), and developed via enhanced chemiluminescence (ECL). Fractions that showed evidence of fH binding by optical densitometry were concentrated using 10,000 MWCO centrifugal filtration units (Amicon, Millipore) then assessed by far-Western analysis with purified fH, as described for dot-blot detection. Protein bands that bound fH were excised from Sypro-ruby stained gels and subjected to mass spectrometry. Non-fractionated cell wall lysates were also assessed for fH binding using purified fH overlay blot. Binding was conducted in 3% BSA/TBST or 3% BSA/TBST containing 0.01 M EDTA.

### Cross-linking fH to *S. aureus*

*S. aureus* Reynolds cells (1×10^9^ cells) were incubated with 10 µg of purified fH for 1 hr in PBS (250 µl total volume) at 37°C. The cross linker BS^3^ (Bis(sulfosuccinimidyl) suberate, Pierce) was added to a final concentration of 50 µM and incubated for 30 mins. After quenching (20 mM Tris-HCl, pH 7.5, 15 mins) and washing, cell wall extracts were prepared as described above. Extracts were subjected to SDS-PAGE and anti-fH Western blot analysis to detect bands that contained fH; samples not incubated with fH were used as controls. Bands determined to contain fH were excised from Sypro-ruby stained gels and assessed by mass spectrometry.

### Mass spectrometry identification

Protein bands were excised from Sypro-ruby stained SDS-PAGE gels and processed for liquid chromatography electrospray ionization tandem mass spectrometry (LC-ESI-MS/MS) as previously described [16]. The acquired data was processed and the proteins were identified using Mascot Daemon client application (Matrix Science) software using an indexed bacterial subset database of the non-redundant proteins database from ExPASy/SwissProt.

### Recombinant proteins

rClfA and rSdrE were expressed as 6×His-tagged proteins comprising their respective unique A regions in an *Escherichia coli* expression system, as described elsewhere [26], [29]. Recombinant proteins were purified from cell lysates by metal chelation chromatography and analyzed via Sypro-ruby stained SDS-PAGE gels and anti His-tag Western blot.

### fH binding to recombinant proteins, dot-blot assay

Briefly, 5 µg of rSdrE, rClfA and BSA were adsorbed to a PVDF membrane. A dilution series of purified fH was also adsorbed for quantitation purposes. The membrane was washed with TBS then blocked with 3% BSA/TBST. Purified fH (20 µg fH/10 ml block buffer) was overlaid overnight. After washing, the membrane was probed with goat anti-fH followed by anti-goat HRP-labeled antibodies (1∶4000 each, Sigma). Membranes were developed via ECL with optical densitometry quantitation via Quantity One software (Biorad).

### fH binding to recombinant proteins, plate assay

Flat-bottom Immulon 2 plates (Thermo Labsystems) were coated with 10 µg/mL of rSdrE, rClfA or BSA in 50 µl of carbonate buffer and incubated overnight at 4°C. Wells were washed to remove unbound protein with PBS with 0.05% Tween (PBST) then blocked with block buffer (3% BSA/PBST for purified fH; 0.5% gelatin/PBST for serum assays) for at least 90 mins at room temperature. Wells were washed, then block buffer containing various amounts of purified fH or serum was added and allowed to incubate at room temperature for the time indicated. Control wells were incubated with block buffer only. Wells were washed, and the presence of fH was assessed using mouse monoclonal anti-fH IgG (1∶500, Serotec) followed by goat anti-mouse-HRP conjugated IgG (1∶1000, Sigma) with 1 hr incubation for each. Plates were developed with TMB substrate (Thermo Scientific), stopped with 1 N H_2_SO_4_, and read at 450 nm. Absorbance values were indicative of fH, with values from wells not incubated with fH or serum subtracted as background.

### fH binding to recombinant proteins, modified plate assay/Western-blot approach

Recombinant proteins were immobilized as described for the plate assay, above. Following blocking with 3% BSA/PBST, 10% HI-serum was added to wells (in block buffer) and incubated overnight. Wells were thoroughly washed, and bound proteins were extracted with 2% SDS buffer and subjected to SDS-PAGE followed by Western blot analysis using goat anti-fH IgG and anti-goat HRP-conjugated IgG (1∶1000 for each, Sigma).

### C3b cleavage to iC3b, plate assay

rSdrE and ovalbumin (50 µL of 30 µg/mL) were adsorbed to microtiter plate wells as described above; ovalbumin (OVA) was used as a control. Wells were blocked with 2% OVA/PBST for 2 hours then purified fH was added and incubated for 30 mins at room temperature. Wells were washed 4 times, then purified C3b and purified fI (0.5 µg each) in 60% DGVB ^++^ (75 µl total volume) were added and plates were incubated for 3 hrs at 37°C. The liquid was extracted and samples were examined for the presence of iC3b by Western blotting probing with goat anti-C3 antibody (1∶4000, CompTech), under reducing conditions. Negative controls included wells not incubated with purified fH while a well coated with goat anti-fH IgG (1∶1000) served as a positive control.

### C3b cleavage to iC3b, *L. lactis*

*L. lactis* (150 µL, OD_600_ = 4.0) were pre-incubated with or without 10% heat-inactivated serum for 20 mins at 30°C in GVBS^++^ to bind serum fH. Bacteria were washed 3× with GVBS^++^, then resuspended in 75 µL of a master mix (300 µL GVBS^++^ with 2 µg purified factor I and 2 µg purified C3b) and incubated at 37°C for 2.5 hr. Purified proteins were purchased commercially from CompTech. The samples were centrifuged and the supernatant was assessed for C3b cleavage via Western blot, under reducing conditions.

### ELISA

For the detection of fH, flat-bottom Immulon 2 plates were coated with 50 µl of goat anti-fH IgG (1∶1000) in carbonate buffer at 4°C, overnight. Following blocking with 3% BSA/PBST (2 hr, room temperature), samples were added to the wells in block buffer (1 hr, room temperature). Unless specified otherwise, probes used were mouse monoclonal anti-fH (Serotec Ltd., Oxford, United Kingdom) with goat anti-mouse HRP-conjugated IgG (Sigma). Probes were incubated for 1 hr each. Purified fH was used as a standard. Plates were developed using TMB substrate, stopped with 1N H_2_SO_4_, and read at 450 nm.

C3 ELISA was performed as described previously using goat anti-C3 IgG (CompTech) to coat; wells were probed with chicken antibodies specific for human C3 (Sigma) and HRP-labeled goat anti-chicken IgG (GenWay) [25]. Purified C3 (CompTech) was used as a standard for quantitation purposes. C5a ELISA was performed as per manufacturer's instructions using the DuoSet ELISA Development kit (R&D systems).

### Serum fH binding to membrane-immobilized *L. lactis*

*L. lactis* expressing SdrE and an empty vector control were resuspended in carbonate buffer to OD_600_ 1.0. Of this mixture, 15 µl was applied to a PVDF membrane, in sets of six per bacterial sample. A dilution series of HI-serum was included for quantitation purposes. The membrane was allowed to dry at 30°C, then blocked with 0.05% gelatin/PBST at room temperature overnight. The membrane was overlaid with 5% HI-serum in 0.05% gelatin/PBST for 1 hr. After washing, the presence of serum fH was assessed using goat anti-fH IgG followed by HRP conjugated anti-goat IgG (1∶4000 each, Sigma). To verify that each bacterial type bound similarly to the membrane, control membranes were washed several times following cell immobilization then stained with Ponceau S and examined using optical densitometry.

### Serum fH binding to *L. lactis* in solution

*L. lactis* were resuspended to OD_600_ = 4.0 in GVBS^++^; 500 µl of the bacterial suspension was incubated with various amounts of HI-serum, in 1 ml total volume, and incubated for 1 hr at 37°C. After washing thoroughly, bound serum proteins were extracted with 50 µl 2% SDS at 95°C for 10 mins. Samples were assessed for serum fH content by Western blotting and fH ELISA using chicken antibodies specific for human fH (Accurate Chemical) followed by goat anti-chicken HRP-conjugated IgG (GenWay).

### C3-fragment deposition

*L. lactis* cells (175 µL, OD_600_ = 4) were incubated with various concentrations of NHS in GVBS^++^, 500 µL total volume, for 15 mins, 37°C. Cells were washed 3× with GVBS-EDTA and bound C3-fragments were stripped using 25 mM methylamine as described previously [25]. To examine C3-fragment deposition from the alternative pathway activation and examine time-dependency, this assay was conducted using GVBS-EGTA with 10% NHS, and incubation times were varied. Total C3-fragment deposition was measured using an ELISA.

### C5a generation

*L. lactis* (175 µL, OD_600_ = 4.0) were incubated with various amounts of NHS in GVBS^++^ at 37°C for 15 mins in 500 µL total volume to generate C5a. To stop the reaction, 100 µL 0.5 M EDTA was added and the supernatant was analyzed for total C5a via ELISA. Alternatively, this assay was conducted using GVBS^++^ EGTA for 30 mins to generate C5a via the alternative pathway.

### Phagocytosis/Killing Assay

Human polymorphonuclear cells (PMNs) were purified from heparinized whole blood from healthy volunteers by Hypaque-Ficoll step gradient centrifugation followed by dextran sedimentation and hypotonic lysis. PMNs were resuspended in HBSS with calcium to 1×10^7^/mL prior to use. *L. lactis* were grown for 3 hrs, resuspended to OD_600_ = 3, then diluted 1∶20 in HBSS with calcium. In 1 mL total volume, 100 µL of bacterial solution, 10% NHS and 5×10^6^ PMNs were tumbled at 37°C. Control tubes did not contain PMNs. Samples were taken at various time points, diluted in sterile water then plated on GM17 agar supplemented with erythromycin. Following an overnight growth at 30°C, colonies were counted. The number of bacteria killed was calculated by subtracting phagocytosis tube counts from control tube counts.

### Statistical analysis

Results of independent replicate experiments were averaged, and SEMs were calculated. Statistical comparisons were made using a two-tailed paired Student's *t* test and ANOVA.

## Results

### *S. aureus* bound fH via a putative fH binding protein (fHbp) associated with the cell wall

**Figure 1:**
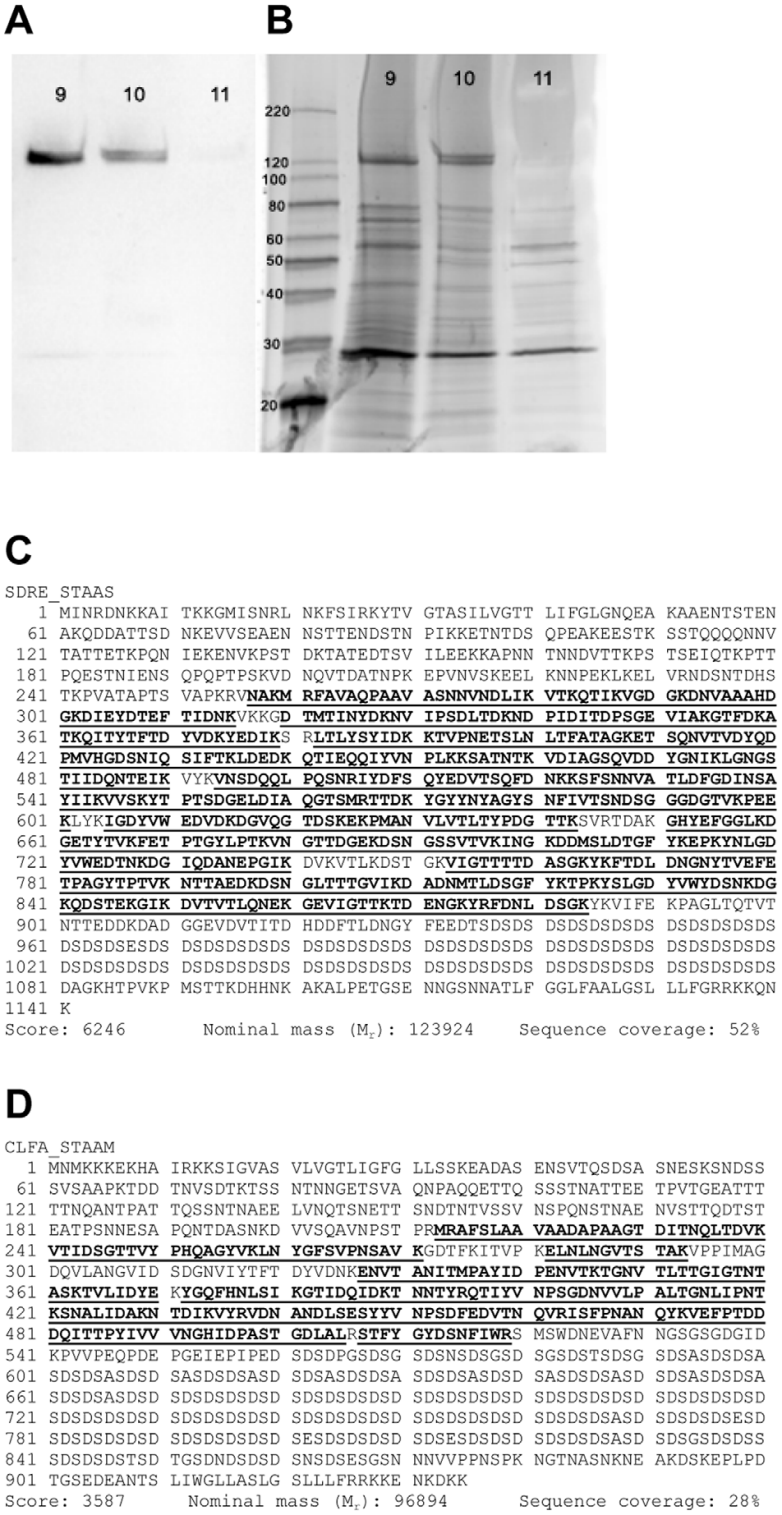
Factor H binds to *S. aureus* cell wall proteins. A, Far-western analysis (purified fH overlay) of *S. aureus* cell wall proteins fractionated via ionic exchange chromatography; 9, 10, and 11 represent fraction numbers. B, Sypro-ruby stained SDS-PAGE gel, complimentary to (A). C, Peptide map of Serine-aspartate repeat-containing protein E (SdrE) with peptides identified by LC-ESI-MS/MS shown in bold and underlined. D, Clumping Factor A (ClfA) peptide map with peptides identified by LC-ESI-MS/MS shown in bold and underlined.

We have previously shown that fH binds to the surface of intact *S. aureus* [25]. To identify the *S. aureus* surface protein responsible for binding fH, we fractionated *S. aureus* cell wall protein preparations by size-exclusion and ionic exchange chromatography. Fractions were adsorbed to a PDVF membrane, blocked, and overlaid with purified fH. Fractions showing evidence of fH binding based on optical densitometry were subjected to SDS-PAGE and far-Western blot analysis (Figure 1A,B).

Protein bands with evidence of fH binding were excised, digested by trypsin, followed by LC-ESI-MS/MS. A database search of identified peptides resulted in the identification of two putative fHbps: SdrE and ClfA (Figure 1C,D). Table 1 details the SdrE and ClfA peptides identified. Peptide scores >30 and Expect scores ≤0.05 are considered significant identifications.

**Figure 2:**
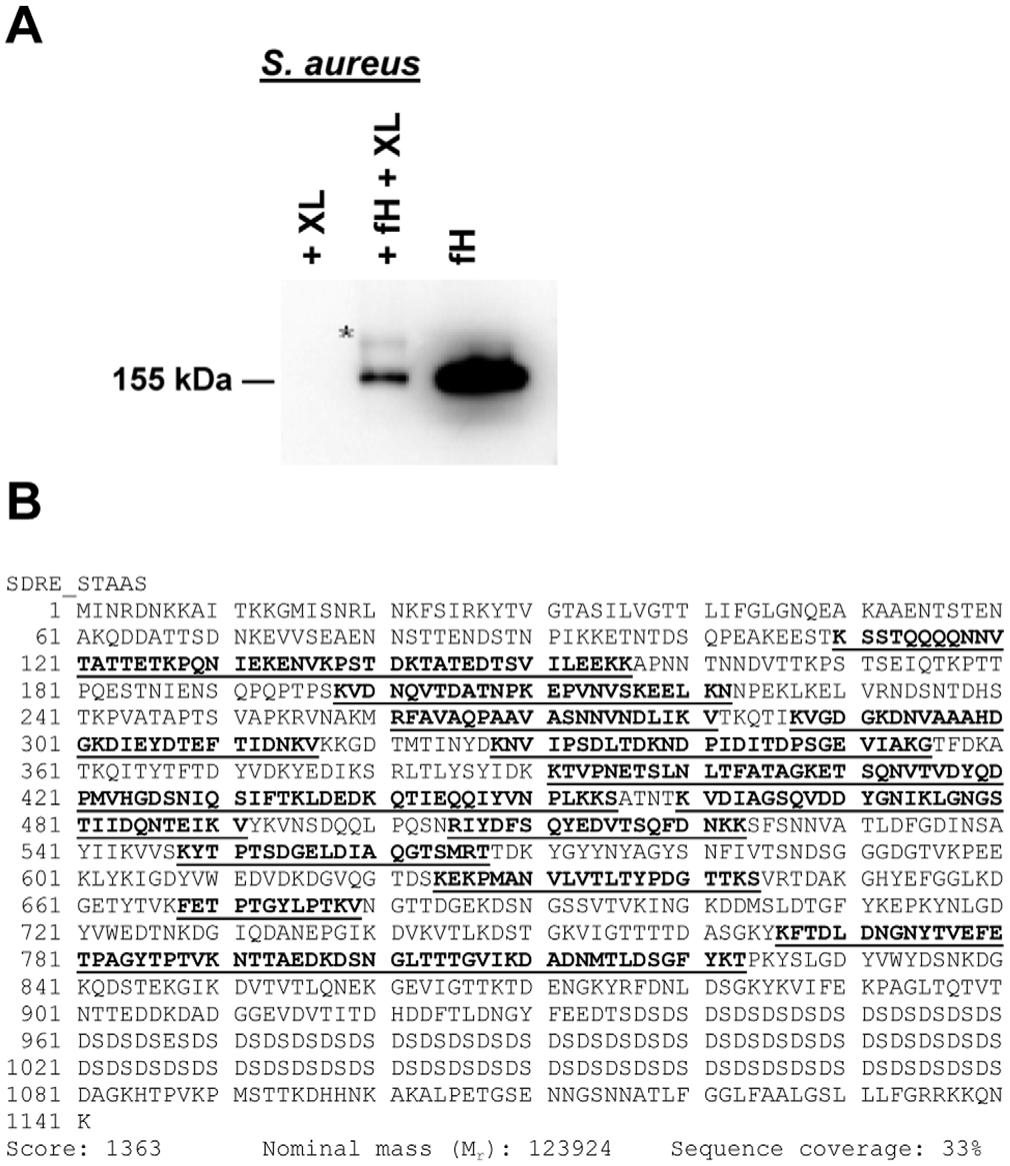
Factor H cross-linked to *S. aureus* cell wall protein/s. A, Anti-fH Western blot of purified fH cross-linked to *S. aureus* cell wall protein/s indicated by the asterisk (*); XL: cross-linker, BS^3^. B, LC-ESI-MS/MS mass spectra map of *S. aureus* protein SdrE found cross-linked to fH. Matched peptides are shown in bold and underlined.

Due to the possibility of the co-migration of SdrE and ClfA, based on their similar molecular weights, we cross-linked purified fH to the *S. aureus* surface with BS^3^, a water-soluble and membrane impermeable crosslinker, followed by Western-blot analysis of solubilized cell wall proteins (Figure 2A). These experiments identified a fH-containing band at an evidently higher molecular weight than purified fH, suggesting fH had been cross-linked to another protein. The novel band was excised and processed by in-gel digestion. LC-ESI-MS/MS analysis of these peptides identified SdrE. The identified peptides and the sequence coverage of the protein are shown in Figure 2B with corresponding peptide data detailed in Table 2. No ClfA peptides were identified.

**Table 1:** Mass spectrometry of fH-binding proteins detected via factor-H overlay identifying both SdrE and ClfA peptides.

SdrE				
M_r_ (expt)	M_r_ (calc)	Peptide score	Expect score	Peptide
894.77	894.41	47	0.0055	R.FDNLDSGK.Y
911.07	911.42	34	0.099	K.DGETYTVK.F
1006.31	1006.49	48	0.0042	K.GHYEFGGLK.D
1114.79	1114.59	62	0.0002	R.LTLYSYIDK.K
1149.27	1149.59	61	0.00027	K.VIGTTTTDASGK.Y
1213.19	1213.57	48	0.0055	K.YRFDNLDSGK.Y
1242.98	1242.69	55	0.0013	R.LTLYSYIDKK.T
1252.86	1252.63	55	0.001	K.FETPTGYLPTK.V
2580.58	2580.16	47	0.0083	K.DNVAAAHDGKDIEYDTEFTIDNK.V
2679.01	2679.22	112	1.7×10^−9^	K.FTDLDNGNYTVEFETPAGYTPTVK.N
2765.79	2765.40	66	9.8×10^−5^	K.NVIPSDLTDKNDPIDITDPSGEVIAK.G
2856.59	2856.26	52	0.0019	K.YNLGDYVWEDTNKDGIQDANEPGIK.D
2970.08	2970.38	79	4.8×10^−6^	K.YKFTDLDNGNYTVEFETPAGYTPTVK.N
3070.79	3070.37	40	0.035	K.ETSQNVTVDYQDPMVHGDSNIQSIFTK.L
3352.57	3352.47	151	1.5×10^−13^	K.YGYYNYAGYSNFIVTSNDSGGGDGTVKPEEK.L
3440.04	3439.55	72	1.9×10^−5^	K.NTTAEDKDSNGLTTTGIKDADNMTLDSGFYK.T
3798.30	3797.69	148	5.7×10^−13^	R.TTDKYGYYNYAGYSNFIVTSNDSGGGDGTVKPEEK.L
3920.30	3920.87	69	3.5×10^−5^	K.GDTMTINYDKVNIPSDLTDKNDPIDITDPSGEVIAK.G

ClfA				
M_r_ (expt)	M_r_ (calc)	Peptide score	Expect score	Peptide
830.62	830.45	53	0.0014	K.SNALIDAK.N
979.67	979.53	47	0.0043	K.TVLIDYEK.Y
1180.74	1180.59	55	0.00062	R.ISFPNANQYK.V
1205.84	1205.62	63	0.00017	K.YGQFHNLSIK.G
1245.33	1245.66	74	1.5×10^−5^	K.ELNLNGVTSTAK.V
1394.97	1394.72	78	5.1×10^−6^	K.LNYGFSVPNSAVK.G
1635.18	1634.85	127	7.8×10^−11^	K.TGNVTLTTGIGTNTASK.T
1638.13	1637.80	61	0.00027	K.GTIDQIDKTNNTYR.Q
1654.99	1654.74	66	6.3×10^−5^	R.STFYGYDSNFIWR.S
1936.08	1935.96	62	0.00016	K.VTIDSGTTVYPHQAGYVK.L
2235.05	2235.07	117	4.6×10^−10^	K.ENVTANITMPAYIDPENVTK.T
2737.43	2737.47	101	1.9×10^−8^	R.QTIYVNPSGDNVVLPALTGNLIPNTK.S
2989.78	2989.32	123	1.6×10^−10^	R.VDNANDLSESYYVNPSDFEDVTNQVR.I
3408.38	3408.54	64	0.00011	K.VYRVDNANDLSESYYVNPSDFEDVTNOVR.I
3569.25	3568.76	138	5×10^−12^	K.VEFPTDDDQITTPYIVVVNGHIDPASTGDLALR.S

**Table 2:** Mass spectrometry of fH-binding protein detected via fH cross-linking identifying SdrE peptides.

M_r_ (expt)	M_r_ (calc)	Peptide score	Expect score	Peptide
1252.17	1252.63	37	0.074	K.FETPTGYLPTK.V
1492.01	1491.62	43	0.021	K.DADNMTLDSGFYK.T
1592.28	1592.77	124	2.1×10^−10^	K.VDIAGSQVDDYGNIK.L
1603.16	1602.81	88	7.9×10^−7^	K.LGNGSTIIDQNTEIK.V
1762.99	1762.91	84	1.4×10^−6^	K.TVPNETSLNLTFATAGK.E
1942.77	1942.02	110	3.6×10^−9^	R.FAVAQPAAVASNNVNDLIK.V
1956.78	1956.87	110	3.9×10^−9^	K.YTPTSDGELDIAQGTSMR.T
1964.10	1963.97	144	1.3×10^−12^	K.NTTAEDKDSNGLTTTGVIK.D
2092.70	2093.07	92	3.4×10^−7^	K.EKPMANVLVTLTYPDGTTK.S
2098.08	2097.92	65	8.2×10^−5^	R.IYDFSQYEDVTSQFDNK.K
2302.41	2302.21	44	0.019	K.LDEDKQTIEQQIYVNPLKK.S
2434.03	2434.20	56	0.00086	K.ENVKPSTDKTATEDTSVILEEK.K
2553.13	2553.29	79	3.6×10^−6^	K.VDNQVTDATNPKEPVNVSKEELK.N
2679.09	2679.22	105	1.3×10^−8^	K.FTDLDNGNYTVEFETPAGYTPTVK.N
2765.96	2765.40	49	0.007	K.NVIPSDLTDKNDPIDITDPSGEVIAK.G
3036.64	3036.39	50	0.004	K.VGDGKDNVAAAHDGKDIEYDTEFTIDNK.V
3069.86	3069.39	64	0.00021	K.ETSQNVTVDYQDPMVHGDSNIQSIFTK.L
3572.74	3572.76	54	0.0012	K.SSTQQQQNNVTATTETKPQNIEKENVKPSTDK.T

### rSdrE binds purified fH in a dose-dependent manner

**Figure 3:**
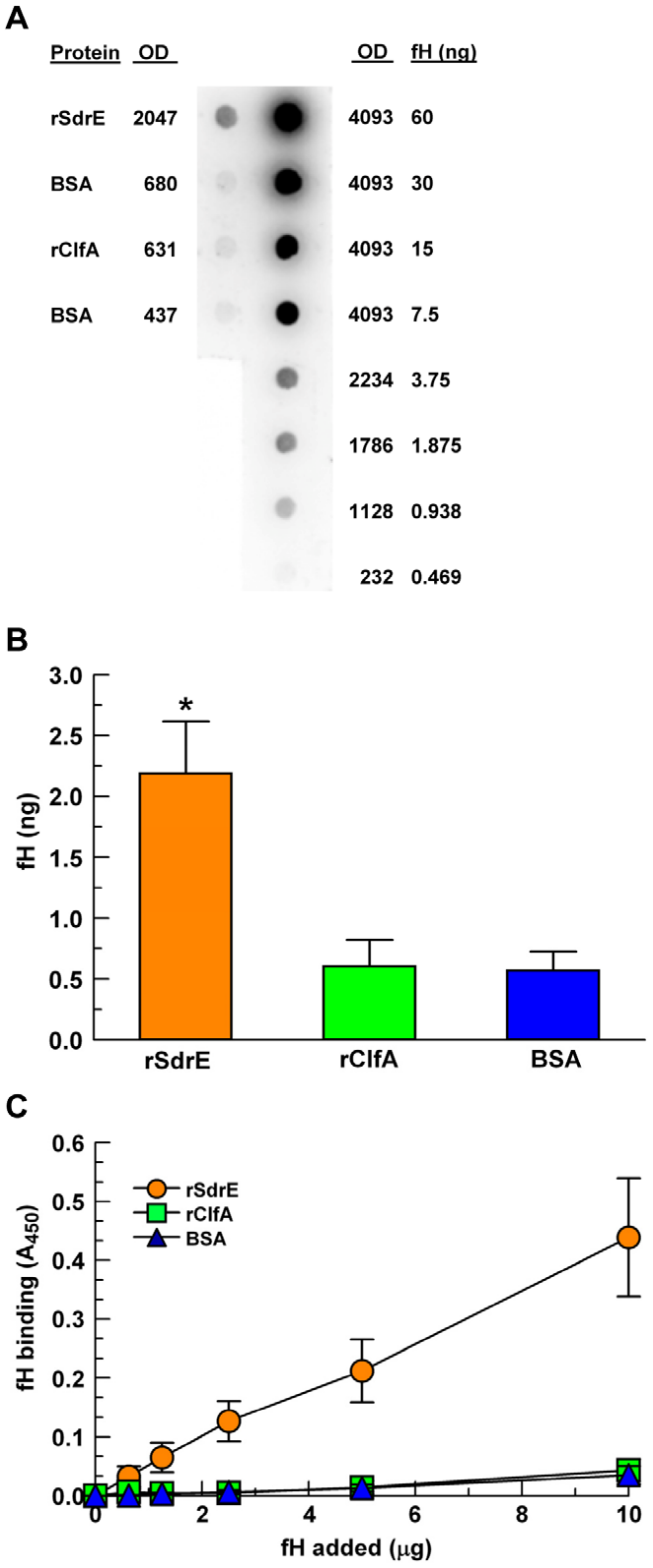
Purified fH binding to recombinant proteins. A, A representative fH overlay dot blot using a purified fH dilution series as a quantitation control (right column, fH). rSdrE, rClfA and BSA (5 µg) were adsorbed to a PDVF membrane (left column), blocked, then overlaid with purified fH (20 µg in 10 ml block buffer); BSA was used as a control. fH binding was determined via optical densitometry using the purified fH dilution series as a standard curve. B, Quantitative fH binding via dot blot, as described in (A), *p<0.01; n = 4. C, Modified ELISA: rSdrE, rClfA and BSA were adsorbed to a microtiter plate and incubated with various amounts of purified fH for 1 hr; data represent the mean of at least three independent experiments ± SEM.

To investigate the fH-binding ability of ClfA and SdrE, we used recombinant forms of the proteins [26], [29]. The purified recombinant proteins and the control protein BSA were adsorbed to a PVDF membrane in equal concentrations (5 µg) and incubated with purified fH. For quantitation of fH binding, we included a dilution series of purified fH to serve as standards. As shown in Figure 3A,B, rSdrE bound 2.2 ng fH, which is >4-fold more than either rClfA or BSA (*p*<0.01).

To further explore the fH binding ability of rSdrE and rClfA, the proteins were adsorbed to the wells of a microtiter plate and incubated with various concentrations of purified fH in an ELISA-type assay. As shown in Figure 3C, rSdrE bound purified fH in a dose-dependent manner, with >10-fold greater binding than either rClfA or the control protein (BSA) (*p* = 0.0019). These results strongly suggest that SdrE is indeed a fHbp.

### rSdrE bound serum fH in a time- and dose-dependent manner

**Figure 4:**
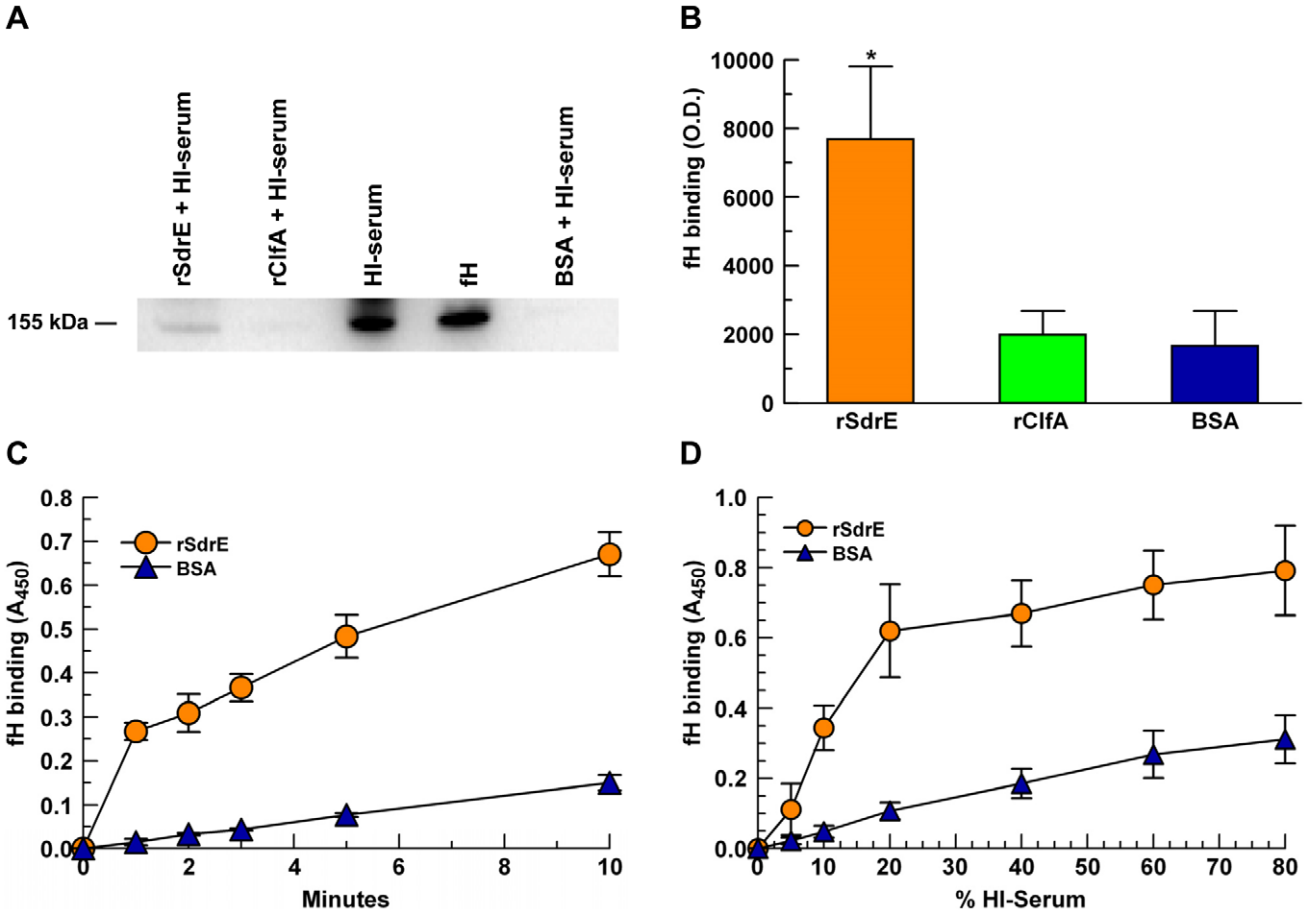
fH binding to recombinant proteins. rSdrE, rClfA and BSA (10 µg/mL) were adsorbed to a microtitre plate and assessed for fH binding using heat-inactivated serum (HI-serum): A, Serum proteins bound to immobilized rSdrE, rClfA, and BSA in microtiter wells were extracted with 2% SDS and analyzed by anti-fH Western blot. B, fH binding via Western blot, as described in (A) quantitated using optical densitometry; *p = 0.03. C, Modified ELISA using 15% HI-serum and various time points; *p*<0.001 as a group. D, Modified ELISA using various concentrations of HI-serum, 15-minute incubation; *p*<0.001 as a group. Data represent the mean of at least three independent experiments ± SEM.

To assess fH binding in more physiological conditions than using purified components, we used heat-inactivated serum as a source of fH. Serum proteins bound to immobilized rSdrE, rClfA and BSA in microtiter wells were extracted and analyzed by anti-fH Western blot (Figure 4A). Optical densitometry readings showed that serum fH binding to rSdrE was 4-fold greater than for rClfA (*p* = 0.03, Figure 4B). However, there was no significant difference between serum fH binding to rClfA compared to BSA control. To further characterize the binding of serum fH to rSdrE, we varied serum concentration and incubation times. As shown in Figure 4C,D, rSdrE bound significantly more serum fH than BSA (*p*<0.001) in a time- and dose-dependent manner.

### rSdrE-bound fH retained regulatory function

**Figure 5:**
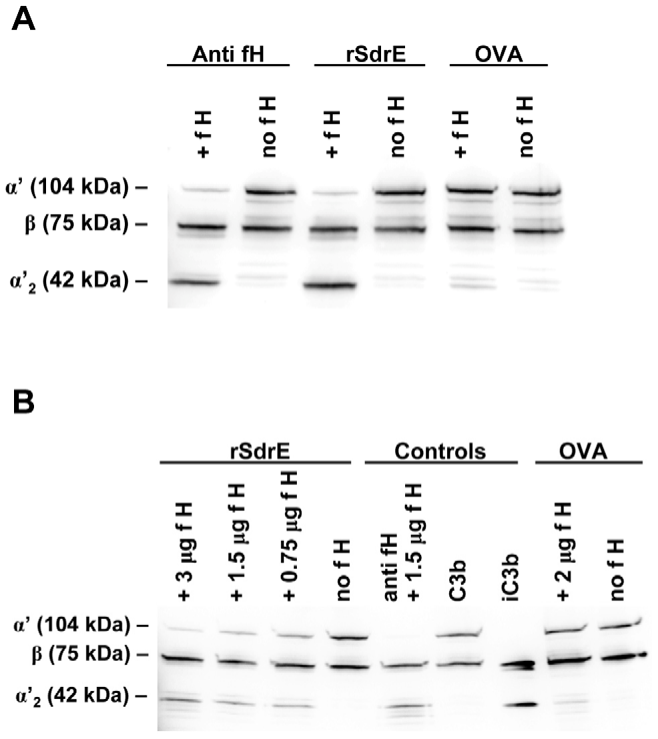
rSdrE-bound fH retains cofactor activity for factor-I mediated cleavage of C3b. rSdrE was immobilized to a microtiter plate and incubated with purified fH; anti-fH and ovalbumin (OVA) were used as controls. After washing, C3b and factor I were added. Well contents were assessed via anti-C3 Western blot for C3b and iC3b. A, Samples with or without 2 µg fH. B, rSdrE with varied amounts of purified fH. Representative Western blots of at least three independent experiments are shown.

To assess whether rSdrE-bound fH retained cofactor functionality for factor I-mediated cleavage of C3b, rSdrE was immobilized to wells of a microtiter plate and incubated with various amounts of purified fH; ovalbumin and goat anti-fH IgG were used as controls. Following washing, purified fI and purified C3b were added. Western blotting revealed that C3b was cleaved to iC3b in wells coated with rSdrE and preincubated with fH (Figure 5A). Increasing concentrations of fH correlated with increasing amounts of C3b cleaved to iC3b (Figure 5B). These experiments suggest that fH bound to rSdrE retains its ability to act as a cofactor for fI in the degradation of C3b.

### *Lactococcus lactis* expressing SdrE bound serum fH in a dose-dependent manner

**Figure 6:**
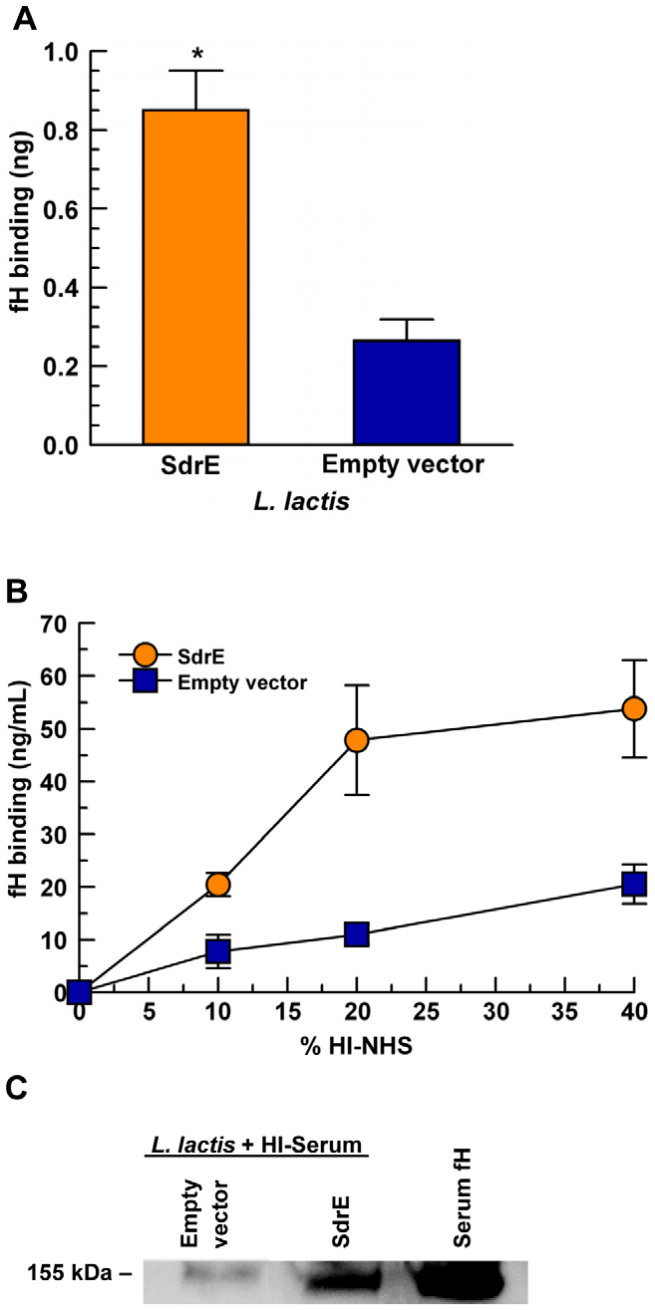
Serum fH binds to SdrE-expressing *L. lactis* in a dose-dependent manner. A, *L. lactis* isogenic mutants were immobilized to a PVDF membrane and overlaid with 5% HI-serum; fH binding was quantitated using a serum fH standard curve and optical densitometry; *p = 0.02. B, *L. lactis* isogenic mutants were incubated with various concentrations of HI-serum for 1 hr; bound proteins were extracted with 2% SDS and analyzed by fH ELISA using chicken antibodies specific for human fH; *p* = 0.005, as a group. C, *L. lactis* were incubated with 20% HI-serum; bound proteins were extracted with 2% SDS and analyzed by anti-fH Western blot. Data represent the mean of at least three independent experiments ± SEM.

To further examine the fH binding ability of SdrE, we used *L. lactis* expressing SdrE. *L. lactis* is a model organism for investigating the individual effects of full-length staphylococcal proteins expressed on the bacterial surface. *L. lactis* (pKS80-SdrE) was compared to the empty vector control *L. lactis* (pKS80). Whole bacteria were immobilized onto a PVDF membrane and overlaid with 5% HI-serum to bind serum fH. Control membranes were processed in parallel, without serum, to assess bacterial adherence. *L. lactis* (pKS80-SdrE) bound 4-fold more serum fH than *L. lactis* (pKS80) (*p* = 0.02), as shown in Figure 6A, while no significant difference in bacterial adherence was detected between groups on control membranes (data not shown).

To further investigate the binding of serum fH to *L. lactis*, we incubated the bacteria with various concentrations of HI-serum and measured serum fH binding to *L. lactis* by ELISA. As shown in Figure 6B, a dose-response relationship exists for *L. lactis* binding of serum fH, with up to 4-fold more serum fH bound to *L. lactis* (pKS80-SdrE) than to *L. lactis* (pKS80) at 20% HI-serum with significantly more binding of serum fH to *L. lactis* (pKS80-SdrE) as a group (*p* = 0.005). Additionally, serum fH extracted from *L. lactis* pre-incubated with 20% HI-serum was analyzed by anti-fH Western blot (Figure 6C). *L. lactis* (pKS80-SdrE) bound much more serum fH than *L. lactis* (pKS80), confirming that surface expression of SdrE enhanced the binding of serum fH to *L. lactis*.

### SdrE-bound fH retains cofactor functionality

**Figure 7:**
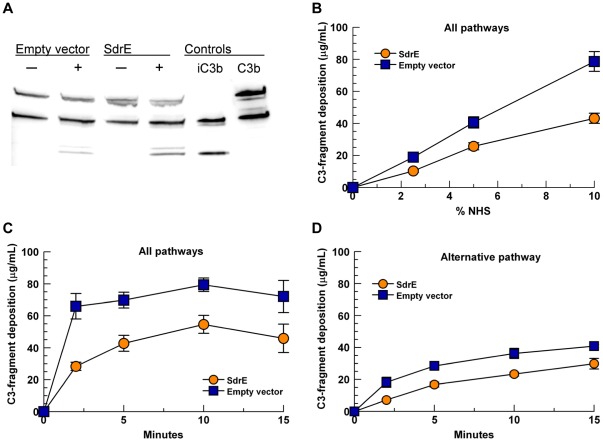
Surface expression of SdrE increases cleavage of C3b and reduces total C3-fragment deposition. A. *L. lactis* were incubated with (+) or without (−) 10% HI-serum to bind serum fH, washed thoroughly, then resuspended with fI and C3b (0.5 µg each) for 2.5 hr, 37°C; supernatants were assessed for C3b cleavage via Western blot. B, C and D: *L. lactis* were incubated with NHS; deposited C3-fragments were stripped with 25 mM methylamine and quantitated by ELISA. B, All pathways, 15-minute incubation, various serum concentrations (*p*<0.001, as a group). C, All pathways, 10% NHS, varied incubation times (*p*<0.0001, as a group). D, Alternative pathway only (Mg-EGTA-GVBS), 10% NHS, various incubation times (*p*<0.0001, as a group). Data represent at least 3 independent experiments ± SEM.

To characterize the significance of fH recruitment by SdrE, we sought to examine whether SdrE-bound fH remained functionally active in its ability to provide cofactor activity for factor I-mediated cleavage of C3b. *L. lactis* variants were incubated with 10% HI-serum to bind serum fH. Following washing, purified C3b and purified factor I were added. Supernatants were examined for C3b cleavage via anti-C3 Western blotting under reducing conditions. As shown in Figure 7A, *L* *. lactis* (pKS80-SdrE) incubated with HI-serum produced more C3b cleavage (iC3b) than the empty vector control, *L. lactis* (pKS80), indicating that fH bound to the bacterial surface via SdrE exhibits cofactor functionality for factor I. Since *L. lactis* (pKS80) binds minimal serum fH, some limited C3b cleavage was evident for this group.

### Surface expression of SdrE reduces total C3-fragment deposition

Since iC3b cannot participate in the formation of an active convertase, thereby preventing additional C3 activation by the alternative pathway, we sought to examine the extent to which the surface expression of SdrE affected C3-fragment deposition on *L. lactis*. *L. lactis* variants were incubated with NHS to allow complement activation via all pathways (GVBS^++^ buffer), as well as the alternative pathway only (Mg-EGTA-GVBS). Surface-bound C3-fragments were stripped with 25 mM methylamine and quantitated by C3 ELISA. In conditions that permitted the activation of all complement pathways, significantly less C3-fragments were deposited on *L. lactis* (pKS80-SdrE) compared to *L. lactis* (pKS80) as a group, using various concentrations of NHS (Figure 7B, *p*<0.001) and varied incubation times (Figure 7C, *p*<0.0001). Under conditions that permitted the activation of the alternative pathway only, a similar result was yielded with significantly less C3-fragment deposition on *L. lactis* (pKS80-SdrE) compared to *L. lactis* (pKS80) as a group (Figure 7D, *p*<0.0001).

### Surface expression of SdrE reduces total C5a generation

**Figure 8:**
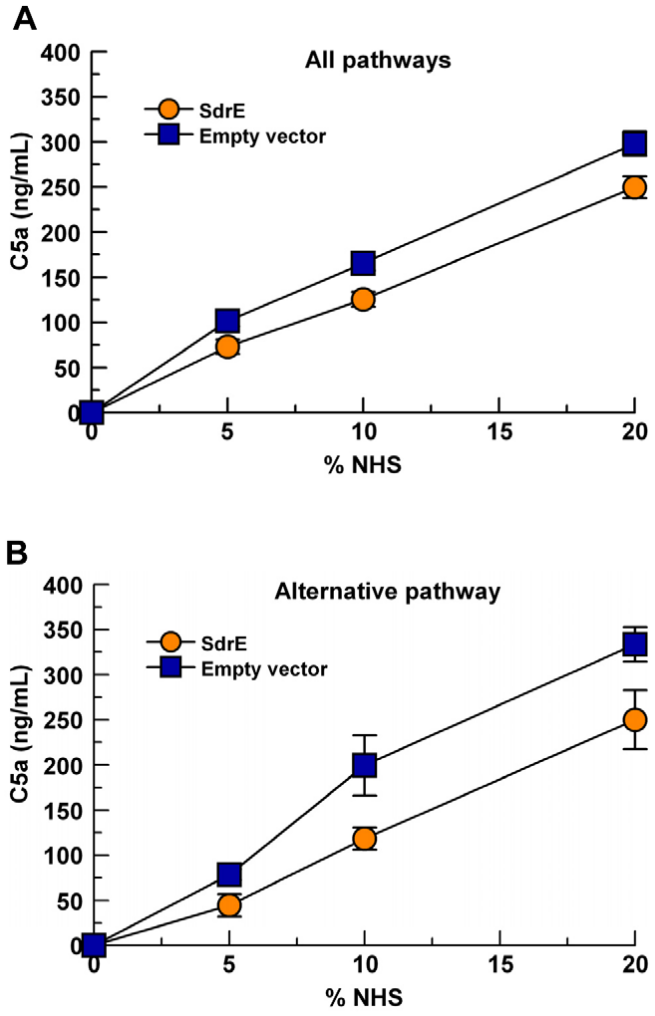
SdrE expression reduces C5a generation. *L. lactis* were incubated with various concentrations of NHS at 37°C. Supernatants were assessed for C5a content via C5a ELISA. A, GVBS^++^ buffer, 15 mins (allows activation of all complement pathways), *p*<0.001, as a group; B, Mg-EGTA-GVBS buffer, 30 mins (alternative pathway activation only), *p*<0.001, as a group. Data represent the mean of 4 independent experiments ± SEM.

Similar to affecting the C3 convertase of the alternative pathway (C3bBb), the cleavage of C3b to iC3b also inhibits the formation of the C5 convertase for all complement pathways as C3b is a component of the C5 convertase for each pathway (C4bC2aC3b, classical and lectin C5-convertase; C3bBbC3b, alternative C5-convertase). Therefore, we examined the effect of SdrE surface expression on the generation of C5a using *L. lactis* variants. *L. lactis* were incubated with various concentrations of NHS for 15 mins in conditions that allowed the activation of all pathways or the alternative pathway only. Complement activation was stopped by the addition of EDTA and supernatants were assessed for evidence of C5a generation by ELISA. As expected, *L. lactis* (pKS80-SdrE) produced significantly less C5a than *L. lactis* (pKS80) in either condition tested as a group (Figure 8A,B, *p*<0.001).

### Surface expression of SdrE reduces the number of bacteria killed by PMNs

**Figure 9:**
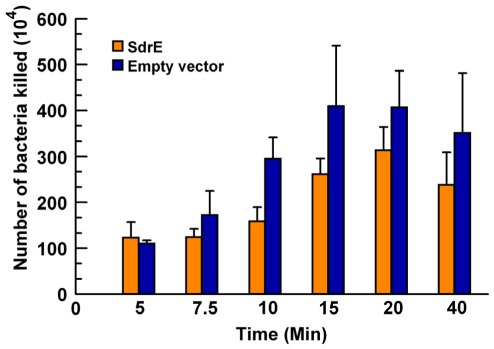
SdrE expression reduces *L. lactis* killing by PMNs. *L. lactis* were incubated with 10% NHS with or without PMNs and tumbled at 37°C. Samples were taken at various time points, diluted in sterile water, and plated (*p* = 0.0016, as a group). Data represent the mean of at least 4 independent experiments ± SEM.

A significant reduction in both C3-fragment opsonization and C5a anaphylatoxin generation indicates a down regulation of complement activation and generation of effectors attributable to the surface expression of SdrE. Therefore, we investigated the extent to which these changes would alter bacterial killing by neutrophils. *L. lactis* variants were incubated with 10% NHS with or without PMNs in conditions that permitted the activation of all complement pathways. As shown in Figure 9, significantly fewer SdrE-expressing *L. lactis* (pKS80-SdrE) were killed than control *L. lactis* (pKS80) as a group (*p* = 0.0016), suggesting that the surface expression of SdrE provides a survival advantage for *L. lactis*.

## Discussion

Many pathogenic bacteria express proteins that interfere with the host defense, with complement evasion a central strategy to their success in causing infection and disease [11], [14], [30], [31], [32], [33]. As a highly successful pathogen, *S. aureus* is no exception. *S. aureus* produces an effective arsenal directly targeted at the host immune system, including the complement cascade and inhibition of its downstream effects. Our current knowledge of the ability of *S. aureus* to subvert the complement system continues to grow.

In the present study, we identified two potential fH-binding *S. aureus* cell wall proteins: SdrE and ClfA. Both belong to the Sdr family of structurally related cell wall-associated proteins that contain a region of serine-aspartate repeats [34], [35], [36]. Anchored via a conserved LPXTG motif, SdrE and ClfA possess an R region containing the repeating SD dipeptides, and a unique A region; SdrE also contains a B region. ClfA is known to bind fibrinogen [34], [37] and fI, as well as function as a cofactor for fI-mediated degradation of C3b [15], [16], [38]. SdrE is less well described; however, it is implicated in human platelet aggregation when expressed on the surface of *Lactococcus lactis* [26]. Until now, a definitive ligand for SdrE has been elusive.

We identified SdrE as a putative fH-binding protein via mass spectrometric analysis of *S. aureus* cell wall proteins isolated by two distinct techniques: purified fH overlay blot of fractionated cell wall proteins as well as cross-linking. The corresponding identifications provided extremely high peptide scores and expect values indicating the strength of these identifications. Studies using recombinantly expressed SdrE validated the ability of this protein to bind fH whether purified or in serum, with a time- and dose-dependent relationship evident. Using a gain-of-function *L. lactis* model, we demonstrated that SdrE expression on a bacterial surface significantly enhances fH recruitment, which confirms our rSdrE-fH binding data. Functional analysis of rSdrE-bound fH revealed that fH remains functionally active in its ability to provide cofactor activity for fI-mediated cleavage of C3b, with a positive correlation of iC3b generation for increasing amounts of fH. This was also observed for *L. lactis*-SdrE-bound fH. Cleaved C3b (iC3b) can no longer participate in the formation of C3- and C5-convertases which negatively affects amplification of the complement cascade [7] resulting in decreased *S. aureus* phagocytosis, as we have previously demonstrated [39]. Likewise, surface expression of SdrE on the surrogate bacterium *L. lactis* resulted in less C3-fragment deposition, less C5a generation, and decreased complement-mediated killing by neutrophils. The down-regulation of complement-mediated host defenses in this gain-of-function model strongly suggests that SdrE is an immune evasion protein. Indeed, *S. aureus* strains that express SdrE are typically associated with invasive infection [40], [41] with 90% of 497 *S. aureus* isolates tested being *sdrE* positive [41].

While characterizing the fH-binding protein band fractionated from cell wall preparations, we also identified ClfA. However, no ClfA peptides were identified in the cross-linked sample. Recombinantly expressed ClfA did not bind purified fH and anti-fH Western-blot analysis of serum proteins bound to rClfA revealed no significant difference between fH binding to rClfA compared to BSA, as determined via optical densitometry. Therefore, it seems reasonable that ClfA was originally identified along with SdrE as a putative fH-binding protein due to co-migration during fractionation and gel electrophoresis attributable to the similar molecular weights and charges of these proteins.

Our earlier examination of *S. aureus*-bound serum proteins suggests that *S. aureus* binds FHL-1, FHR-1α, and/or FHR-1β [25]. Therefore, it is possible that these proteins interact with SdrE. However, under the conditions tested, we were unable to demonstrate binding of FHL-1, FHR-1α, and/or FHR-1β to recombinant SdrE or SdrE expressed on the surface of *L. lactis*. The serum concentration of FHL-1 is 10–50 times lower than fH [40], which may have contributed to the negative result. FHR-1α and FHR-1β are known to be at a much lower concentration in serum than fH; however, their concentrations have not been clearly defined [41]. Therefore, whether FHL-1 and/or FHR-1α/β interact with SdrE will be further addressed in future studies.

The staphylococcus protein Sbi has previously been shown to bind fH in a triparte complex with C3b via Sbi domains III and IV [42]. Sbi can be found in both the cytoplasmic membrane fraction as well as secreted into the external milieu [43]. The N-terminal domains of Sbi (I and II) bind IgG in a similar manner to staphylococcal protein A when exposed on the cell surface, whereas the C-terminal domains of Sbi (III and IV) are only biologically active when secreted [43]. Therefore, Sbi cannot contribute to the acquisition of fH to the staphylococcal surface.

In summary, our data show that rSdrE is a clear fH-binding molecule due to its ability to bind fH whether purified or in serum. Moreover, rSdrE-bound fH retains cofactor activity for fI-mediated cleavage of C3b. Additionally, our studies using SdrE-expressing *L. lactis* demonstrate that SdrE expression on the bacterial surface increases the binding of fH, down-regulates complement effectors, and provides protection from neutrophil killing. As such, SdrE recruitment of fH likely provides a survival advantage for *S. aureus* by negatively affecting the formation of complement activating complexes, thereby dampening the host immune response. To our knowledge, this is the first description of a *S. aureus* surface protein that recruits the potent complement regulator fH to evade the immune response.
